# Effectiveness of uterine tamponade devices for refractory postpartum haemorrhage after vaginal birth: a systematic review

**DOI:** 10.1111/1471-0528.16819

**Published:** 2021-07-19

**Authors:** V Pingray, M Widmer, A Ciapponi, GJ Hofmeyr, C Deneux, M Gülmezoglu, K Bloemenkamp, OT Oladapo, D Comandé, A Bardach, P Vázquez, G Cormick, F Althabe

**Affiliations:** ^1^ Instituto de Efectividad Clínica y Sanitaria (IECS‐CONICET) Buenos Aires Argentina; ^2^ UNDP‐UNFPA‐UNICEF‐WHO‐World Bank Special Program of Research, Development, and Research Training in Human Reproduction (HRP) Department of Sexual and Reproductive Health and Research World Health Organization Geneva Switzerland; ^3^ University of Botswana Gaborone Botswana; ^4^ Effective Care Research Unit University of the Witwatersrand/Walter Sisulu University Mthatha South Africa; ^5^ Obstetrical Perinatal and Pediatric Epidemiology Research Team EPOPé INSERM INRA U1153 Centre for Epidemiology and Statistics Sorbonne Paris Cité (CRESS) Université de Paris Paris France; ^6^ Concept Foundation Geneva Switzerland; ^7^ Department of Obstetrics Birth Centre Wilhelmina's Children Hospital, Division Woman and Baby University Medical Center Utrecht Utrecht The Netherlands

**Keywords:** Bakri balloon, condom uterine balloon tamponade, hysterectomy, maternal death, postpartum haemorrhage, uterine atony, vaginal delivery

## Abstract

**Objectives:**

To evaluate uterine tamponade devices’ effectiveness for atonic refractory postpartum haemorrhage (PPH) after vaginal birth and the effect of including them in institutional protocols.

**Search strategy:**

PubMed, EMBASE, CINAHL, LILACS, POPLINE, from inception to January 2021.

**Study selection:**

Randomised and non‐randomised comparative studies.

**Outcomes:**

Composite outcome including surgical interventions (artery ligations, compressive sutures or hysterectomy) or maternal death, and hysterectomy.

**Results:**

All included studies were at high risk of bias. The certainty of the evidence was rated as very low to low. One randomised study measured the effect of the condom‐catheter balloon compared with standard care and found unclear results for the composite outcome (relative risk [RR] 2.33, 95% CI 0.76–7.14) and hysterectomy (RR 4.14, 95% CI 0.48–35.93). Three comparative studies assessed the effect of including uterine balloon tamponade in institutional protocols. A stepped wedge cluster randomised controlled trial suggested an increase in the composite outcome (RR 4.08, 95% CI 1.07–15.58) and unclear results for hysterectomy (RR 4.38, 95% CI 0.47–41.09) with the use of the condom‐catheter or surgical glove balloon. One non‐randomised study showed unclear effects on the composite outcome (RR 0.33, 95% CI 0.11–1.03) and hysterectomy (RR 0.49, 95% CI 0.04–5.38) after the inclusion of the Bakri balloon. The second non‐randomised study found unclear effects on the composite outcome (RR 0.95, 95% CI 0.32–2.81) and hysterectomy (RR 1.84, 95% CI 0.44–7.69) after the inclusion of Ebb or Bakri balloon.

**Conclusions:**

The effect of uterine tamponade devices for the management of atonic refractory PPH after vaginal delivery is unclear, as is the role of the type of device and the setting.

**Tweetable abstract:**

Unclear effects of uterine tamponade devices and their inclusion in institutional protocols for atonic refractory PPH after vaginal delivery.

## Tweetable abstract

Unclear effects of uterine tamponade devices and their inclusion in institutional protocols for atonic refractory PPH after vaginal delivery.

## Introduction

Haemorrhage continues to be the most significant direct cause of maternal death, accounting for 661 000 deaths worldwide between 2003 and 2009.^1^ Most of these deaths occur during the immediate postpartum period and are due to uterine atony, a condition characterised by the failure of the uterus to contract adequately after the delivery of the placenta.^2^

Most women with postpartum haemorrhage (PPH) respond well to first‐line interventions (uterotonics, uterine massage, tranexamic acid). However, 10–20% are unresponsive to these interventions – a subgroup (denoted as ‘refractory PPH’) where most of the PPH‐related morbidity and mortality are concentrated.^3^ Between one‐third and one‐half of refractory PPH cases are due to uterine atony. Laparotomy for compressive sutures, ligation of uterine blood supply or hysterectomy is frequently needed to prevent deaths among these women.^4^, ^5^ Embolisation of uterine arteries by interventional radiology is also an option, although availability in low‐resource settings is very limited.^2^

Effective non‐surgical interventions to manage refractory PPH are critical to avoid surgical treatment and to provide treatment in settings in which surgical treatment is not available. Surgical interventions are associated with an increased risk of severe morbidity and mortality and are not widely available in low‐resource settings. The non‐surgical interventions currently recommended by the World Health Organization (WHO) for the treatment of refractory PPH due to uterine atony include manual compressive measures (bimanual uterine compression and external aortic compression), uterine balloon tamponade (UBT) and a second dose of tranexamic acid.^2^, ^6^

### Description of the intervention

Under the umbrella of uterine tamponade devices for treating refractory PPH, two categories were considered: uterine balloon tamponade (UBT) devices and uterine suction tamponade (UST) devices. Briefly, UBTs consist of inserting a rubber, silicone or plastic balloon into the uterine cavity and inflating the balloon with a sterile liquid.^7^ The inflated balloon exerts outward pressure on the uterus, achieving a tamponade effect to prevent further bleeding.^8^ The UBT can be administered using either improvised or purpose‐designed devices.^9^ Improvised devices encompass balloon catheters designed for other purposes and used off‐label to treat PPH (i.e. the Sengstake–Blakemore tube, the Rusch balloon, the Foley catheter), as well as those based on the use of condoms and surgical gloves attached to Foley or other catheters. Purpose‐designed UBTs for PPH treatment are the Bakri^®^ balloon, the EBB^®^ tamponade system (Belfort‐Dildy), the Ellavi balloon (by Sinapi Biomedical) and the BT‐Cath^®^ balloon.^2^, ^7^, ^10^, ^11^

More recently, a novel type of device that uses vacuum force to retract the uterus has been proposed as an alternative to the UBT.^12^ Such USTs could be considered a physiologically plausible alternative for the management of unresponsive PPH, as the mechanism of action mimics physiological uterine retraction. Similar to UBT, there are UST purpose‐designed and improvised devices.^8^, ^13^

### Why it is important to do this review

The previous WHO recommendation on UBT was based on case series and studies with no control population, leading to a conditional recommendation. This conditional recommendation does not support the widespread application of UBT in all clinical situations. Since the WHO recommendation was published, several additional studies have been reported, including randomised controlled trials (RCTs). Given the importance of UBT as a potentially life‐saving intervention and the popularity of the intervention globally, it is relevant to systematically review all data available to date, including these newer studies’ findings.

The proliferation of UBT devices over the years, with variable rates of success in reducing PPH‐related morbidity, demands a careful assessment of reported tamponade devices to determine their comparative effectiveness and safety. We undertook the present systematic review aiming to address two key objectives: (1) to evaluate the clinical effectiveness and safety of different uterine tamponade devices used for the treatment of atonic refractory PPH following vaginal birth, compared with any non‐surgical intervention (e.g. pharmacological and mechanical treatments) administered for the treatment of PPH; and (2) to evaluate the effect of including uterine tamponade devices in an institutional protocol for the management of refractory PPH following vaginal birth.

## Methods

This systematic review was conducted following a protocol specifically designed for this purpose and reported according to the PRISMA statement’s recommendations (Table S1). The protocol was registered in PROSPERO (CRD42019120486).

### Selection of studies

For the first objective, eligible studies were randomised or non‐randomised studies that evaluated a uterine tamponade device’s effectiveness versus standard care in women who developed atonic refractory PPH after vaginal birth (individual‐level interventions). For the second objective, randomised and non‐randomised studies with a control group or period that evaluated the effect of including uterine tamponade devices in institutional protocols for the treatment of refractory PPH, compared with the use of protocols without tamponade devices (facility‐level intervention) were included. Abstracts were eligible if sufficient data were reported.

### Outcomes

Primary outcomes were: (1) a composite outcome including surgical interventions (laparotomy for artery ligations, uterine compressive sutures or hysterectomy) or maternal death, and (2) hysterectomy.

Secondary outcomes were: conservative surgical interventions (compressive sutures and/or artery ligations), maternal death, shock, coagulopathy, organ dysfunction, blood transfusion, transfer to a higher level of care, women’s sense of wellbeing, acceptability of and satisfaction with the intervention, initiation of breastfeeding and other adverse effects.

The selected outcomes are consistent with those suggested by the Core Outcome Set initiative.^14^ We excluded studies that did not report any of the outcomes previously listed.

### Search strategy

The search strategy was developed with a librarian’s assistance in electronic search strategies for systematic reviews (Appendix S1).

The search was run from inception to January 2021 in the following electronic databases: PubMed, EMBASE, CINAHL, LILACS and POPLINE. The search was complemented by reviewing the references of all articles selected for full‐text reading and by looking for unpublished studies through contacts with investigators who are experts in the field. There were no language restrictions. We sought out translations if studies were not reported in English, French and Spanish (languages spoken by reviewers). If translations were not found, then language restrictions were applied.

### Data extraction and synthesis

Citations were downloaded from the reference manager RIS to covidence,^15^ a web‐based platform used to support the conduct of systematic reviews. Titles and abstracts of all imported citations were screened by at least two reviewers using covidence, and those that were potentially eligible were selected for full‐text review. At least two independent reviewers performed the process of study selection and data extraction (MW, VP, GC). A form designed explicitly for this review was used to extract data from the included studies. Disagreements were discussed until consensus was reached, and a third reviewer was consulted if required. Where information from an article was not clear, its authors were contacted to provide additional details.

### Risk of bias assessment

Two reviewers assessed the risk of bias by using the ‘Risk of bias’ tool described in the *Cochrane Handbook for Systematic Reviews of Interventions* for randomised studies and the ROBINS‐I tool (Risk of Bias in Non‐Randomised Studies of Interventions) for non‐randomised studies.^16^, ^17^ For randomised studies, random sequence generation and allocation concealment, were assessed at the study level. The following were assessed at the outcome level: blinding of participants and personnel and outcome assessors; incomplete outcome data, selective reporting; other bias. Quality assessment criteria used to assess non‐randomised studies were: bias due to confounding, bias in the selection of participants into the study, bias in classification of interventions, bias due to deviations from intended interventions, bias due to missing data, bias in the measurement of outcomes, bias in the selection of the reported result and overall bias. We assessed the risk of bias for each criterion as 'low risk', 'high risk' and 'unclear risk' (Table S2 and Table S3).

In addition, the Grading of Recommendations Assessment, Development and Evaluation (GRADE) Criteria^18^ were used to assess the certainty of the evidence for the outcomes prioritised in this review. The overall certainty in the evidence was classified in one of four categories: high, moderate, low or very low.

### Strategy for analysis and data synthesis

Studies assessing individual‐level interventions were analysed with the number of all women with PPH after vaginal birth as the denominator, but the studies evaluating facility‐level interventions were analysed with the total number of vaginal births as the denominator. This is because facility‐level interventions could affect PPH detection rates. Hence, the most comparable populations between periods or hospitals are all women having vaginal births during the study periods.

As all variables from which data could be obtained were found to be dichotomous, we calculated risk ratios (RR) with 95% CI. Two out of four included studies reported outcomes using a different denominator or measure of effect. Whenever possible, we conducted additional pre‐specified subgroup analyses by type of device (purpose‐designed and improvised devices) and by setting: low‐ and middle‐income countries (LMICs) and high‐income countries (HICs). The summary statistics for each of the included studies are reported in tables. Given the variations in denominators and measures of effect, the summary table includes effect estimates reported by each study’s authors. Meta‐analyses were not possible because of high degrees of clinical heterogeneity. Cochrane's review manager 5.3^19^ software was used to conduct statistical analyses.

## Results

### Description of studies

The search strategy yielded a total of 10 650 citations. After screening titles and abstracts, the reviewers selected 538 citations for full‐text review. Twenty‐six studies were eligible according to our selection criteria. Four out of 26 compatible citations were ultimately included^20^, ^21^, ^22^, ^23^ (Figure S1). The excluded studies and the reasons for exclusion are described in Table S4. No studies assessed the effectiveness of UST devices. Included studies were published between January 2007 and October 2019.

Table 1 presents the main characteristics of the four included studies. One study conducted in Benin and Mali assessed UBT devices’ effectiveness for refractory postpartum haemorrhage after vaginal birth by comparing the condom‐catheter balloon against standard care.^22^ Three studies evaluated the effects at the facility‐level of including UBT devices as a treatment option for refractory PPH after vaginal birth, including one stepped‐wedge cluster RCT conducted in Uganda, Senegal and Egypt introducing condom or glove catheter^20^ and two non‐randomised studies conducted in France: one comparing outcome rates at the hospital‐level before and after the introduction of the Bakri balloon^21^ and the other comparing outcomes between one perinatal network using the Bakri/EBB^®^ and one control network.^23^

**Table 1:** Main characteristics of included studies for the evaluation of the effectiveness

Research question	Study design	Study and year	Country	Sample size	Inclusion criteria	Intervention	Control	Main outcome
Q1. Any type of uterine tamponade device vs standard care (individual‐level intervention)	Randomised	Dumont et al. 2017	Benin and Mali	116	PPH due to suspected uterine atony unresponsive to first‐line treatment after vaginal delivery	Condom‐catheter balloon + misoprostol	Misoprostol	Surgical intervention (arterial ligatures, uterine compressive sutures, hysterectomy) or death before discharge
Q2. Inclusion of UBT in an institutional protocol for the management of PPH compared with protocols without UBT (facility‐level intervention)	Randomised	Anger et al. 2019	Uganda, Senegal and Egypt	59 765	Vaginal delivery; delivery at a study hospital or referral to a study hospital for PPH after delivery elsewhere	Condom‐catheter balloon or surgical glove	Standard care	Maternal death or invasive procedures
	Non‐randomised	Laas et al. 2012	France	23 863	PPH due to uterine atony that is unresponsive to sulprostone after a vaginal delivery or caesarean section	Bakri balloon	Oxytocin and sulprostone	Arterial embolisation, conservative surgical procedures (artery ligations and/or uterine compression sutures), and hysterectomy
	Non‐randomised	Revert et al. 2018	France	73 529	Women with PPH from uterine atony unresponsive to sulprostone after a vaginal delivery or a caesarean section	Bakri or ebb balloon	Medical treatment	Arterial embolisation or surgery (pelvic vessel ligation or hysterectomy)

**Figure 1:**
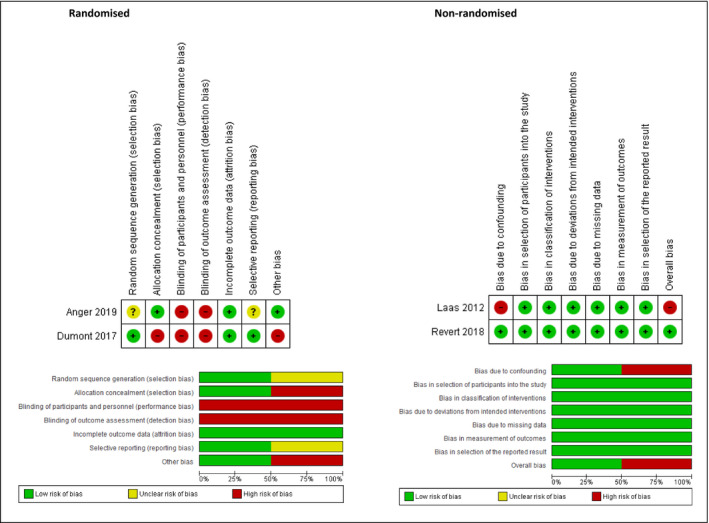
Quality assessment of included studies.

To assess the included studies’ validity, we rated individual criteria for each study, which were specific for randomised and non‐randomised studies. Details of the quality of each individual study are described in Figure 1 and Table S5. Overall, the studies showed a high risk of bias.

In concordance with the Cochrane Agency for Healthcare Research and Quality standards, these studies were rated as low‐quality randomised trials. Although the included non‐randomised studies were judged as high‐to‐moderate quality, they carry the biases inherent to their respective study designs.

### Effect of the interventions

#### Effect of any type of uterine tamponade device versus standard care in women with refractory PPH

Table 2 shows the effect of any type of UBT device versus no device in women with atonic refractory PPH (individual‐level intervention) on primary and secondary outcomes. Only one RCT reported the effect of the use of a condom‐catheter balloon on these outcomes.^22^ There is an unclear risk of surgical interventions or death associated with the use of the condom‐catheter balloon plus misoprostol compared with misoprostol alone (RR 2.33, 95% CI 0.76–7.14). The same RCT^22^ reported unclear results for hysterectomy (RR 4.14, 95% CI 0.48–35.93). For the secondary outcomes, the results of this trial are unclear and graded as very low‐certainty evidence (risk of conservative surgical interventions (RR 2.07, 95% CI 0.54–7.88), maternal death (RR 6.21, 95% CI 0.77–49.98), blood transfusions (RR 1.49, 95% CI 0.88–2.51) and transfer to a higher level of care (RR 1.29, 95% CI 0.55–3.04).

**Table 2:** Summary of findings for the first comparison: intrauterine balloon tamponade compared with standard care for the management of refractory PPH (individual‐level intervention)

Outcomes	Study	Relative effect (95% CI)	Certainty of the evidence
Composite outcome (surgical interventions and/or death)	Dumont et al. 2017	RR 2.33 (0.79–7.14)	⨁⨁◯◯ Low^a,b^
Hysterectomy to control bleeding	Dumont et al. 2017	RR 4.14 (0.48–35.93)	⨁◯◯◯ Very low^a,c^
Conservative surgical interventions (CS and/or, AL)	Dumont et al. 2017	RR 2.07 (0.54–7.88)	⨁⨁◯◯ Low^a,b^
Maternal death due to bleeding	Dumont et al. 2017	RR 6.21 (0.77–49.98)	⨁◯◯◯ Very low^a,c^
Blood transfusion	Dumont et al. 2017	RR 1.49 (0.88–2.51)	⨁⨁◯◯ Low^a,b^
Transfer to a higher level of care	Dumont et al. 2017	RR 1.29 (0.55–3.04)	⨁⨁◯◯ Low^a,b^

Explanations: ^a^ Downgraded one level because of the high risk of bias on blinding, other bias (imbalanced baseline) and unclear allocation concealment; ^b^ Downgraded one level because of its wide confidence interval; ^c^ Downgraded two levels because of its too wide confidence interval.

AL, artery ligation; CS, compressive sutures.

The risk in the intervention group is based on the assumed risk in the comparison group and the relative effect of the intervention (and its 95% CI). GRADES of evidence: High certainty: we are very confident that the true effect lies close to that of the effect's estimate. Moderate certainty: we are moderately confident in the effect estimate: the true effect is likely to be close to the effect's estimate, but there is a possibility that it is substantially different. Low certainty: our confidence in the effect estimate is limited: the true effect may be substantially different from the effect's estimate. Very low certainty: we have very little confidence in the effect estimate: the true effect is likely to be substantially different from the estimate of effect.

Subgroup analyses by device or setting were not possible. The included RCT evaluated an improvised device and was conducted in Benin and Mali, two low‐income countries.^22^

#### Effect of including UBTs in institutional protocols versus either a previous period in which the UBT was not used or other clinical settings without including UBT

The effects of including UBT devices in institutional protocols for the treatment of refractory PPH on primary and secondary outcomes are shown in Table 3.

**Table 3:** Summary of findings for the second comparison: use of intrauterine balloon tamponade as part of an institutional protocol for the management of refractory PPH (facility‐level intervention)

Outcome	Study	Effect estimate (95% CI)		Certainty of the evidence (for the effect estimate among all vaginal births)
		All vaginal births as denominator	Reported by study authors	
Composite outcome (surgical interventions and/or death)	Anger et al. 2019	RR^a^ 4.08 (1.07–15.58)	RR^a^ 4.08 (1.07–15.58)	⨁⨁◯◯ Low^d^, ^e^
	Laas et al. 2012	RR 0.33 (0.11–1.03)	Not reported	⨁⨁◯◯ Low^g^
	Revert et al. 2018	RR^b^ 0.95 (0.32–2.81)	RR^a^ 0.14 (0.08–0.27)	⨁⨁◯◯ Low^g^
Hysterectomy	Anger et al. 2019	RR^a^ 4.38 (0.47–41.09)	RR^a^ 4.38 (0.47–41.09)	⨁◯◯◯ Very low^e^, ^g^
	Laas et al. 2012	RR 0.49 (0.04–5.38)	OR^c^ 0.44 (0.04–4.91)	⨁◯◯◯ Very low^d^, ^g^
	Revert et al. 2018	RR 1.84 (0.44–7.69)	Not reported	⨁◯◯◯ Very low^d^, ^g^
Conservative surgical interventions (CS, AL)	Anger et al. 2019	RR 2.82 (1.03–7.71)	RR 2.82 (1.03–7.71)	⨁⨁◯◯ Low^d^, ^e^
	Laas et al. 2012	RR 0.29 (0.08–1.06)	OR^c^ 0.26 (0.07–0.95)	⨁⨁◯◯ Low^g^
	Revert et al. 2018	RR 0.21 (0.02–1.82)	Not reported	⨁◯◯◯ Very low^d^, ^g^
Maternal death	Anger et al. 2019	RR^a^ 2.23 (0.35–14.07)	RR^a^ 2.23 (0.35–14.07)	⨁◯◯◯ Very low^e^, ^f^
	Laas et al. 2012	No events	No events	–
	Revert et al. 2018	Cannot estimate	Not reported	–
Blood transfusion	Anger et al. 2019	RR^a^ 1.24 (0.86–1.80)	RR^a^ 1.24 (0.86–1.80)	⨁⨁◯◯ LOW^d^, ^e^
	Laas et al. 2012	RR 1.43 (0.76–2.71)	OR^c^ 1.31 (0.67–2.56)	⨁◯◯◯ VERY LOW^d^, ^g^
	Revert et al. 2018	Not reported	Not reported	—
Transfer–higher level of care	Anger et al. 2019	RR^a^ 3.05 (0.79–11.70)	RR^a^ 3.05 (0.79–11.70)	⨁◯◯◯ VERY LOW^e^, ^f^
	Laas et al. 2012	Not reported	Not reported	—
	Revert et al. 2018	Not reported	Not reported	—

The effect estimate for the composite outcome reported by the authors in Revert *et al*. 2018 includes artery embolisations. CI: Confidence interval; RR: Risk ratio; **GRADE Working Group grades of evidence: High certainty:** We are very confident that the true effect lies close to that of the effect's estimate. **Moderate certainty:** We are moderately confident in the effect estimate: The true effect is likely to be close to the effect's estimate, but there is a possibility that it is substantially different. **Low certainty:** Our confidence in the effect estimate is limited: The true effect may be substantially different from the effect's estimate. **Very low certainty:** We have very little confidence in the effect estimate: The true effect is likely to be substantially different from the estimate of effect.

AL, artery ligation; CS, compressive sutures.

a Adjusted.

b In contrast to the composite outcome reported by the study authors, we did not include artery embolisation in the composite outcome for this review.

c Study authors used the number of women who required intravenous sulprostone as the denominator.

d Downgraded one level due to its wide confidence interval.

e Downgraded one level due to high risk of bias on blinding, and unclear risk of bias on random sequence generation and selective reporting.

f Downgraded two levels due to its wide confidence interval.

g Downgraded two levels because the included studies are non‐randomised studies.

The experimental study by Anger et al., which used a stepped‐wedge design, suggests a four‐fold statistically significant increase in surgical interventions or maternal deaths associated with introducing improvised UBTs (RR 4.08, 95% CI 1.07–15.58).^20^ In contrast, two non‐randomised studies showed unclear effects on the composite outcome after the inclusion of the Bakri balloon (RR 0.33, 95% CI 0.11–1.03) and Bakri/Ebb balloon (RR 0.95, 95% CI 0.32–2.81).^21^, ^23^

Three studies reported hysterectomy rates and were graded as low‐certainty evidence. The Anger et al. trial used the condom‐catheter device and found unclear results (RR 4.38, 95% CI 0.47–41.81),^20^ as did both non‐randomised studies that assessed purpose‐designed UBT (RR 0.49, 95% CI 0.04–5.38 and RR 1.84, 95% CI 0.44–7.69, respectively).^21^, ^23^

Regarding the subsequent need for conservative surgical interventions (artery ligation, compressive sutures), the RCT (Anger et al.) suggests a statistically significant increase in the risk of additional conservative interventions associated with improvised devices (RR 2.82, 95% CI 1.03–7.71),^20^ whereas the non‐randomised studies evaluating purpose‐designed devices showed unclear results (RR 0.29, 95% CI 0.08–1.06^21^, ^23^ and RR 0.21, 95% CI 0.02–1.82^23^). For other secondary outcomes, the RCT assessing the condom‐catheter device found unclear results for maternal deaths (RR 2.23, 95% CI 0.35–14.07), blood transfusion (RR 1.24, 95% CI 0.86–1.80) and transfer to a higher level of care (RR 3.05, 95% CI 0.79–11.70).^20^ Neither of the non‐randomised studies assessing purpose‐designed UBTs provided additional data regarding maternal death; no maternal deaths due to PPH were reported in the Laas study, and the risk of maternal death after vaginal delivery was not assessed in the Revert et al. study.^21,23^ Laas et al. reported unclear results on blood transfusions (RR 1.43 95% CI 0.76–2.71). Neither of the non‐randomised studies reported the effect of a purpose‐designed device on transfer to a higher level of care.

The study by Revert et al. considered artery embolisation in its primary outcome (a composite outcome of surgical interventions), and the authors conducted the analysis and interpretation of the results on that basis.^23^ As we did not include invasive non‐surgical interventions among the surgical interventions in our primary outcome, we analysed the Revert et al. study data excluding women receiving such a procedure. The results of this study, including artery embolisation in the composite outcome as reported by the authors, show a statistically significant reduction in the surgical interventions and deaths associated with the use of UBTs (adjusted RR 0.14, 95% CI 0.08–0.27), whereas unclear results were found when excluding artery embolisation (RR 0.95, 95% CI 0.32–2.81).

It was not possible to analyse effects by device or setting. The Anger et al. trial evaluating an improvised device was conducted in LMICs, whereas the non‐randomised studies evaluating purpose‐designed devices were conducted in HICs.

Some of the outcomes of interest, such as blood loss, shock, coagulopathy, organ dysfunction, women’s sense of wellbeing, acceptability of and satisfaction with the intervention, and breastfeeding, were not reported in the included studies.

## Quality of the evidence according to GRADE assessment

Tables 2 and 3 show details on the quality of evidence according to GRADE criteria for the two comparisons of interest. Overall, the assessment showed a low to very low certainty of the evidence for all outcomes. For the first comparison – any type of uterine tamponade devices compared with no devices – we found low quality of evidence for the composite outcome and very low quality for hysterectomy in the study evaluating the use of UBT at the individual level. Similar judgements were obtained (low quality of evidence for the composite outcome and very low quality for secondary outcomes) for the second comparison – inclusion of uterine tamponade devices in institutional protocols – when evaluating purpose‐designed devices at the facility‐level, independent of the study design. The quality of evidence was low to very low for all secondary outcomes: hysterectomy, surgical interventions, maternal death, blood transfusion and transfer to a higher level of care. These results were consistent across different study designs (randomised and non‐randomised) and level of intervention (individual or facility).

## Discussion

### Summary of main results

Four studies assessing the effectiveness and safety of UBTs for the treatment of atonic refractory PPH after vaginal delivery were included. The evidence from the RCT^22^ assessing the effect of improvised UBT devices in women with refractory PPH showed unclear results in subsequent surgical interventions, maternal deaths or hysterectomy alone when compared with standard care. Three studies assessing the effect of including UBTs in an institutional protocol for PPH management showed conflicting results. The RCT^20^ suggested an increase in the composite of subsequent surgical interventions and maternal deaths and unclear results in the risk of hysterectomy associated with the use of the condom‐catheter or surgical glove balloon. The two non‐randomised studies assessing the inclusion of purpose‐designed balloons in institutional protocols found an unclear effect on the composite outcome and hysterectomy^21^, ^23^. Although the RCTs evaluated the improvised UBTs in LMICs, the non‐randomised studies assessed purpose‐designed UBTs and were conducted in HICs. Therefore, it was not possible to disentangle the effect by type of device or by setting.

### Overall completeness, quality of the studies and quality of the evidence

After a detailed quality assessment of the studies included in this systematic review, we identified substantive methodological flaws in both RCTs and determined that they had a ‘high’ risk of bias. The included non‐randomised studies were judged as high‐to‐moderate quality but had the biases inherent to their respective study designs. Consequently, for the systematic review of primary outcomes, the certainty of the evidence was graded as low to very low due to study limitations and because of imprecision.

### Factors that may be determinants of the effect of UBT

#### Improvised UBTs versus purpose‐designed UBTs

One randomised trial comparing the condom‐catheter to the Bakri balloon reported a longer time to control bleeding with the condom‐catheter balloon but no difference in substantive outcomes.^24^ In addition, further analysis suggests that implementation fidelity and quality may influence findings. For example, the studies using improvised devices in LMICs reported delays in treatment administration. The Dumont et al. trial^22^ reported that the condom‐catheter balloon was inserted within 30 minutes of PPH diagnosis in only 58% of cases. Furthermore, the stepped‐wedge cluster RCT by Anger et al. mentioned that providers reported a problem with the condom‐catheter balloon in 52% of cases.^20^

#### The setting

The effective management of refractory PPH requires an expedited stepwise approach, in which the availability of resources and a well‐operating health system are essential.^25^ It is plausible that in settings where the identification of PPH and subsequent quality of care are more likely to be substandard, the effect of the UBT may be different from in settings with good availability of resources and high quality of care. The Dumont et al. trial reported that frequent delays in the diagnosis and treatment of uterine atony were observed, with a high proportion of women receiving a late injection of oxytocin in the first response.^22^ Similarly, the stepped‐wedge cluster RCT by Anger et al.^20^ reported that blood shortages were a problem for almost half of PPH‐related deaths in the study, including some cases in which, despite bleeding stopping after administration of the UBT, the woman did not recover because timely blood replacement was unavailable. The authors suggested that, ‘interventions such as UBT may have limited effectiveness in improving maternal outcomes when introduced into resource‐constrained health systems with unreliable access to other essential components of emergency care’.^20^

Another potentially important aspect of the setting is whether the UBT procedure is performed in the delivery room or the surgical theatre. Typically, in some HICs, like the UK and the USA, the procedure is conducted in the surgical theatre, following uterine cavity exploration to exclude trauma as the cause of the bleeding. Conversely, in LMICs, the procedure is usually performed in the delivery room, frequently without exploring the uterine cavity. On the one hand, performing the procedure in the surgical theatre after excluding other causes may avoid applying the UBT in cases with no uterine atony, so avoiding delays to administering the correct treatment. Additionally, if the UBT fails, surgical treatment can be started without delay.

On the other hand, in low‐resource settings, such requirements may contribute to the delay of the UBT procedure. In the Dumont et al. trial, a large proportion of the UBT procedures were performed in the operating theatre of referral hospitals. The authors reported ‘the recurring unavailability of the theatre had an important consequence in the delays for the experimental group’.^22^

### Strengths and limitations

The strengths of this systematic review include following rigorous Cochrane methods and the PRISMA protocol for reporting. The broad search strategy captured a large number of published and unpublished studies. To assess effectiveness, we tightly restricted eligibility to studies that selected women with suspected uterine atony and refractory PPH and reported additional surgical interventions or maternal death. We included all types of studies that compared the effectiveness of UBT with medical treatment and the local standard of care. Case reports were not included to assess effectiveness; given that this systematic review will inform clinical and policy decision‐making, comparative effectiveness evidence is required. Although this review’s inclusion time frame was intentionally long to identify a wide range of devices reported in the literature, most included studies were published recently. As the included studies used different types of UBT devices and were conducted in different countries, an effort was made to highlight these distinctions throughout the analysis.

Our review also has some limitations mainly derived from the scarcity and kind of information reported in articles. We found very few studies reporting the effect of UBT in atonic refractory PPH after vaginal delivery. We excluded 13 analytical studies because outcomes were measured in all births,^26^, ^27^, ^28^, ^29^, ^30^, ^31^, ^32^, ^33^, ^34^, ^35^, ^36^, ^37^, ^38^ without disaggregating data according to the mode of delivery (Table S4), with one‐quarter to one‐half of the included cases ending in caesarean sections. Six studies were excluded due to: insufficient data,^39^, ^40^ involved women having a caesarean section,^41^ the intervention being administered as part of a package,^42^ UBT being administered as a first‐line treatment for PPH,^43^ the outcomes reported differing from the prioritised outcomes in this systematic review^44^, ^45^, ^46^ or involving the comparison of two different types of UBT.^24^ It was possible to extract data after vaginal birth in only two studies.^21^, ^23^ Finally, the inability to pool risk estimates due to the heterogeneity in the study designs should be noted. The heterogeneity in the estimation of blood loss and the definition of refractory PPH is also a limitation of this study.

### Agreements and disagreements with other reviews

We compared our findings with other systematic reviews published after 2017 when the first RCTs were published.^47^, ^48^, ^49^ In 2020, Suarez et al. published a comprehensive systematic review, including RCTs, non‐randomised studies of interventions, and case series that reported on the efficacy, effectiveness, and/or safety of UBT in women with PPH due to a variety of PPH aetiologies after vaginal or caesarean delivery.^47^ The primary outcome was the UBT success – defined as bleeding arrested without maternal death or additional surgical or radiological interventions in women in which the UBT was placed. The present systematic review differs from that of Suarez et al. in that we did not include case report studies, given their key limitation of not having a comparison group. Additionally, we restricted our focus to atonic refractory PPH after vaginal delivery only. Both reviews acknowledge the conflicting evidence and unclear results from RCTs compared with non‐randomised studies. However, Suarez et al. primarily based their conclusions on the results observed in the uncontrolled studies and suggested that the evidence of benefit is persuasive.

Moreover, the authors stated that the priority is to evaluate delivery strategies for the introduction of UBT through implementation research, suggesting that no more effectiveness research is needed. This is consistent with a previous commentary published in 2018 by the same group proposing that it was time for global scale‐up and not RCTs of UBT.^50^ Our systematic review included only well‐controlled studies, which was likely to be a fundamental factor in our less enthusiastic findings.

In 2020, Kellie et al. published a Cochrane systematic review of mechanical and surgical interventions to treat primary PPH. The review included only RCTs, both in PPH after vaginal birth or after caesarean section. Specifically, about uterine tamponade devices, the authors concluded that there is currently insufficient evidence from RCTs to determine their effectiveness and safety.^48^ Finally, in 2019, Ali et al. published a systematic review on studies using the Bakri balloon. Based mainly on uncontrolled observational studies, the authors concluded that the Bakri balloon showed ‘little effectiveness’ and that more RCTs should be conducted.^49^

## Conclusion

According to the body of evidence currently available, the effect of UBT for the management of atonic refractory PPH after vaginal delivery is unclear. Whether the type of device or the setting is important, factors associated with UBTs’ effect is unknown.

### Implications for practice

There is uncertainty about the effectiveness and safety of UBT for the treatment of women with refractory PPH after vaginal delivery in low‐resource settings with unreliable access to good‐quality PPH care. Acknowledging that, our view is that UBT should be considered for routine refractory PPH care in settings where birth attendants are appropriately trained to use tamponade devices and manage PPH, where access to surgical interventions and blood products are available if needed, where the differential diagnosis of other causes of PPH can be performed, and where the resources required for PPH management are routinely available, and maternal status can be appropriately monitored. In facilities currently using UBT that do not meet these criteria, our view is that it should be considered only in the context where the use of an unproven method is clinically thought to be better than other available alternatives, and keeping in mind the need not to delay other definitive interventions such as surgical or up‐referral.

### Implications for research

In low‐resource settings, the efficacy and safety of UBT for the treatment of women with refractory PPH after vaginal delivery should be evaluated through good‐quality RCTs in well‐functioning health systems. In well‐resourced settings, it is a priority to assess the comparative efficacy of different purpose‐designed UBTs against improvised devices.

### Disclosure of interests

VP, MW, FA, AC, GC, CD, MG, OTO, VPa, AB and DC have no conflicts of interests. GJH initiated the use of the Levin suction catheter as a uterine suction tamponade device. He did not participate in decisions regarding the inclusion of reports on the Levin tube method in the review. Completed disclosure of interests form is available to view online as supporting information.

### Contribution to authorship

Study conceptualisation was by FA and MW. FA, MW, GC, AC and VP contributed to drafting the protocol. MW, GC, AB and VP selected studies for inclusion and extracted data. DC designed and ran the search strategy. DC and PVa located all full texts. AC, AB and VP conducted the quality assessments. AC and VP conducted data analysis. FA, MW, VP, AC and GC contributed to drafting the review. VP, MW, AC, GC, KB, CD, MG, GJH, OTO, VPa, AB, DC and FA reviewed, provided comments and edits, and approved the manuscript.

### Funding

Funding was provided by UNDP/UNFPA/UNICEF/WHO/World Bank Special Programme of Research, Development and Research Training in Human Reproduction, Department of Sexual and Reproductive Health and Research, WHO.

### Acknowledgements

We thank Thomas Allen for developing the search strategy and Ayodele Lewis and Caitlin R. Williams for editing the manuscript.

## Supporting information


**Figure S1**. Study flow diagram.

**Table S1**. Prisma checklist.

**Table S2**. Cochrane risk of bias tool for studies.

**Table S3**. Assessment tool for judging non‐randomised studies (ROBINS‐I).

**Table S4**. Randomised and non‐randomised studies excluded from the quantitative synthesis.

**Table S5**. Overall methodological quality of included RCTs and non‐RCTs.

**Appendix S1**. Search strategy.

Supplementary Material

Supplementary Material

Supplementary Material

## Data Availability

The data that support the findings of this study are available from the corresponding author upon reasonable request.
