# Supplementary material for: Effectiveness of uterine tamponade devices for refractory postpartum haemorrhage after vaginal birth: a systematic review
Source: BJOG. 2021 Jul 19;128(11):1732–43. doi: 10.1111/1471-0528.16819 (PMC9292664; doi:10.1111/1471-0528.16819)
Supplement: Supplementary file 1 — Figure S1. Study flow diagram. [file BJO-128-1732-s002.docx]

**SF 1: Study flow diagram**

Duplicates (n = **874**)

Records identified through database searching

(n = **10650**)

Irrelevant studies

(n = **9238**)

Records screened

(n = **9776**)

Full-text articles non-eligible studies excluded

(n = **512**)

**233** Ineligible study design

**102** Wrong intervention

**77** Ineligible patient population

**41** Duplicate

**23** missing full text

**10** Language restriction

**8** Ongoing study

Full-text articles assessed for eligibility

(n = **538**)

Excluded from the analysis

(n = **22**)

**14** Not possible to extract data on women with vaginal birth

**4** Insufficient data

**1** Intervention administrated as a package

**1** UBT applied as first-line treatment

**1** Compared to another UBT treatment

**1** Not reporting prioritized outcomes

Comparative eligible studies

(n = **26**)

Studies included in the analysis

(n = **4**)
