# Supplementary material for: Effectiveness of uterine tamponade devices for refractory postpartum haemorrhage after vaginal birth: a systematic review
Source: BJOG. 2021 Jul 19;128(11):1732–43. doi: 10.1111/1471-0528.16819 (PMC9292664; doi:10.1111/1471-0528.16819)
Supplement: Supplementary file 3 — Table S2. Cochrane risk of bias tool for studies. [file BJO-128-1732-s007.docx]

**Table S2: COCHRANE RISK OF BIAS TOOL FOR STUDIES**

| Criteria | Description |
| --- | --- |
| Random sequence generation | Score “Low risk” if a random component in the sequence generation process is described (e.g. Referring to a random number table). Score “High risk” when a non-random method is used (e.g. performed by date of admission). Non-randomised trials and controlled before-after studies should be scored “High risk”. Score “Unclear risk” if not specified in the paper. |
| Allocation concealment | Score “Low risk” if the unit of allocation was by institution, team or professional and allocation was performed on all units at the start of the study; or if the unit of allocation was by patient or episode of care and there was some form of centralised randomisation scheme, an on-site computer system or sealed opaque envelopes were used. Controlled before-after studies should be scored “High risk”. Score “Unclear risk” if not specified in the paper. |
| Baseline outcome measurements similar | Score “Low risk” if performance or patient outcomes were measured prior to the intervention, and no important differences were present across study groups. In randomised trials, score “Low risk” if imbalanced but appropriate adjusted analysis was performed (e.g. Analysis of covariance). Score “High risk” if important differences were present and not adjusted for in analysis. If randomised trials have no baseline measure of outcome, score “Unclear risk”. |
| Baseline characteristics similar | Score “Low risk” if baseline characteristics of the study and control providers are reported and similar. Score “Unclear risk” if it is not clear in the paper (e.g. characteristics are mentioned in text but no data were presented). Score “High risk” if there is no report of characteristics in text or tables or if there are differences between control and intervention providers. Note that in some cases imbalance in patient characteristics may be due to recruitment bias whereby the provider was responsible for recruiting patients into the trial. |
| Incomplete outcome data | Score “Low risk” if missing outcome measures were unlikely to bias the results (e.g. the proportion of missing data was similar in the intervention and control groups or the proportion of missing data was less than the effect size i.e. unlikely to overturn the study result). Score “High risk” if missing outcome data was likely to bias the results. Score “Unclear risk” if not specified in the paper (Do not assume 100% follow up unless stated explicitly). |
| Knowledge of the allocated interventions adequately prevented during the study | Score “Low risk” if the authors state explicitly that the primary outcome variables were assessed blindly, or the outcomes are objective, e.g. length of hospital stay. Primary outcomes are those variables that correspond to the primary hypothesis or question as defined by the authors. Score “High risk” if the outcomes were not assessed blindly. Score “Unclear risk” if not specified in the paper. |
| Protection against contamination | Score “Low risk” if allocation was by community, institution or practice and it is unlikely that the control group received the intervention. Score “High risk” if it is likely that the control group received the intervention (e.g. if patients rather than professionals were randomised). Score “Unclear risk” if professionals were allocated within a clinic or practice and it is possible that communication between intervention and control professionals could have occurred (e.g. physicians within practices were allocated to intervention or control) |
| Selective outcome reporting | Score “Low risk” if there is no evidence that outcomes were selectively reported (e.g. all relevant outcomes in the methods section are reported in the results section). Score “High risk” if some important outcomes are subsequently omitted from the results. Score “Unclear risk” if not specified in the paper. |
| Other risks of bias | Score “Low risk” if there is no evidence of other risk of biases. |
