# Supplementary material for: Effectiveness of uterine tamponade devices for refractory postpartum haemorrhage after vaginal birth: a systematic review
Source: BJOG. 2021 Jul 19;128(11):1732–43. doi: 10.1111/1471-0528.16819 (PMC9292664; doi:10.1111/1471-0528.16819)
Supplement: Supplementary file 4 — Table S3. Assessment tool for judging non‐randomised studies (ROBINS‐I). [file BJO-128-1732-s001.docx]

**Table S3: ASSESSMENT TOOL FOR JUDGING NON-RANDOMIZED STUDIES (ROBINS-I)**

| Domain | Explanation |
| --- | --- |
| **Pre-intervention** | **Risk of bias assessment is mainly distinct from assessments of randomised trials** |
| Bias due to confounding | Baseline confounding occurs when one or more prognostic variables (factors that predict the outcome of interest) also predict the intervention received at baseline. ROBINS-I can also address time-varying confounding, which occurs when individuals switch between the interventions being compared and when post-baseline prognostic factors affect the intervention received after baseline. |
| Bias in selection of participants into the study | When exclusion of some eligible participants, or the initial follow-up time of some participants, or some outcome events is related to both intervention and outcome, there will be an association between interventions and outcome even if the effects of the interventions are identical. This form of selection bias is distinct from confounding—A specific example is bias due to the inclusion of prevalent users, rather than new users, of an intervention. |
| **At intervention** | **Risk of bias assessment is mainly distinct from assessments of randomised trials** |
| Bias in classification of interventions | Bias introduced by either differential or non-differential misclassification of intervention status. Non-differential misclassification is unrelated to the outcome and will usually bias the estimated effect of intervention towards the null. Differential misclassification occurs when misclassification of intervention status is related to the outcome or the risk of the outcome, and is likely to lead to bias. |
| **Post-intervention** | **Risk of bias assessment has substantial overlap with assessments of randomised trials** |
| Bias due to deviations from intended interventions | Bias that arises when there are systematic differences between experimental intervention and comparator groups in the care provided, which represent a deviation from the intended intervention(s). Assessment of bias in this domain will depend on the type of effect of interest (either the effect of assignment to intervention or the effect of starting and adhering to intervention). |
| Bias due to missing data | Bias that arises when later follow-up is missing for individuals initially included and followed (such as differential loss to follow-up that is affected by prognostic factors); bias due to exclusion of individuals with missing information about intervention status or other variables such as confounders. |
| Bias in measurement of outcomes | Bias introduced by either differential or non-differential errors in measurement of outcome data. Such bias can arise when outcome assessors are aware of intervention status, if different methods are used to assess outcomes in different intervention groups, or if measurement errors are related to intervention status or effects. |
| Bias in selection of the reported result | Selective reporting of results in a way that depends on the findings and prevents the estimate from being included in a meta-analysis (or other synthesis). |
