# Supplementary material for: Effectiveness of uterine tamponade devices for refractory postpartum haemorrhage after vaginal birth: a systematic review
Source: BJOG. 2021 Jul 19;128(11):1732–43. doi: 10.1111/1471-0528.16819 (PMC9292664; doi:10.1111/1471-0528.16819)
Supplement: Supplementary file 5 — Table S4. Randomised and non‐randomised studies excluded from the quantitative synthesis. [file BJO-128-1732-s009.docx]

**Table S4: Randomised and non-randomised studies excluded from the quantitative synthesis**

| **Type of study** | **Reason** | **Study** |
| --- | --- | --- |
| *Randomised* | The outcomes reported differ from the prioritised outcomes in this systematic review. | ^1^ |
|  | It addresses a different research question. This study evaluated the effect of UBT as a first-line treatment (not refractory PPH) | ^2^ |
|  | Insufficient data reported. The total sample size is reported, but the number of participants per group is not reported. | ^3^ |
|  | Compare UBT (improved-design) to another UBT (purpose-designed | ^4^ |
|  | Outcomes include all deliveries. Not possible to extract data on women with vaginal deliveries only. | ^5-17^ |
| *Non-randomised* | Uterine tamponade device used after caesarean sections. | ^18^ |
|  | The effect of UBT cannot be measured since the intervention was administered as a package that included UBT and NASG. | ^19^ |
|  | The outcomes reported differ from the prioritised outcomes in this systematic review. | ^20,21^ |
|  | Insufficient data reported. The total number of women undergoing a vaginal birth is not reported. | ^22^ |

**References**

(1) Nomia A, Afroze A, Kiran K. Efficacy and Safety of Intrauterine Balloon Tamponadeversus Uterovaginal Roll Gauze Packing in Patient Presenting with Primary Postpartum Hemorrhage after Normal Vaginal Delivery.

(2) Soltan MH, Mohamed A, Ibrahim E, Gohar A, Ragab H. El-menia air inflated balloon in controlling atonic post partum hemorrhage. International journal of health sciences 2007;1(1):53-9.

(3) El Gelany, S. A. A., Soltan MH. External aortic compression device, manual aortic compression & El Minya air inflated balloon: simple, cost-effective, and saving many lives in low resource settings. International journal of gynaecology and obstetrics 2012;119:S335.

(4) Darwish AM, Abdallah MM, Shaaban OM, Ali MK, Khalaf M, Sabra AMA. Bakri balloon versus condom-loaded Foley's catheter for treatment of atonic postpartum hemorrhage secondary to vaginal delivery: a randomised controlled trial. Journal of Maternal-Fetal & Neonatal Medicine 2018;31(6):747-753.

(5) Houlihan C, Virk K, Lowe W, Dhillon P, Guzman E. The impact of the Bakri Balloon on the rate of cesarean hysterectomy at a single university hospital. Obstet Gynecol 2013;208(1):S59.

(6) Von Beckerath AK, Maul H, Elmohandes AM, Shaaban M, Habib DM, Nasr A, et al. Comparison of celox and bakri balloon in management of primary atonic postpartum hemorrhage. American journal of obstetrics and gynecology 2016;214(1):S335.

(7) Lo A, St. Marie P, Yadav P, Belisle E, Markenson G. The impact of Bakri balloon tamponade on the rate of postpartum hysterectomy for uterine atony. Journal of Maternal-Fetal & Neonatal Medicine 2017;30(10):1163-1166.

(8) Patane L, Cavalli G, Mandelli V, Strobelt N, Frigerio L, Pirola S, et al. Bakri balloon tamponade and uterine packing with gauze in post partum hemorrhage management: Any differences? Obstet Gynecol 2014;210(1):S322.

(9) Cornelissen L, Woodd S, Shakur-Still H, Fawole B, Noor S, Etuk S, et al. Secondary analysis of the WOMAN trial to explore the risk of sepsis after invasive treatments for postpartum hemorrhage. Int J Gynaecol Obstet 2019 Aug;146(2):231-237.

(10) Dueckelmann AM, Hinkson L, Nonnenmacher A, Siedentopf JP, Schoenborn I, Weizsaecker K, et al. Uterine packing with chitosan-covered gauze compared to balloon tamponade for managing postpartum hemorrhage. Eur J Obstet Gynecol Reprod Biol 2019 Sep;240:151-155.

(11) Kong CW, To WWK. Menstrual and reproductive outcome after use of balloon tamponade for severe postpartum haemorrhage. J Perinat Med 2017;45:361.

(12) Ramler PI, Henriquez DDCA, van den Akker T, Caram-Deelder C, Groenwold RHH, Bloemenkamp KWM, et al. Comparison of outcome between intrauterine balloon tamponade and uterine artery embolisation in the management of persistent postpartum hemorrhage: A propensity score-matched cohort study. Acta Obstet Gynecol Scand 2019 Nov;98(11):1473-1482.

(13) Gauchotte E, Torre DL, Perdriolleâ€Galet E, Lamy C, Gauchotte G, Morel O. Impact of uterine balloon tamponade on the use of invasive procedures in severe postpartum hemorrhage. Acta Obstet Gynecol Scand 2017;96(7):877-882.

(14) Mishra N, Gulabani K, Agrawal S, Shrivastava C. Efficacy and Feasibility of Chhattisgarh Balloon and Conventional Condom Balloon Tamponade: A 2-Year Prospective Study. J Obstet Gynaecol India 2019 Oct;69(Suppl 2):133-141.

(15) Rozenberg P, Sentilhes L, Winer N, Goffinet F, Vayssiere C, Senat M, et al. 485 Efficacy of early versus late intrauterine balloon tamponade in the management of severe postpartum hemorrhage. Obstet Gynecol 2021;224(2):S308-S308.

(16) Seasely AR, Szychowski JM, Saxon N, Casey BM, Tita A, Subramaniam A. 805 Vacuum-induced hemorrhage control versus balloon tamponade for management of postpartum hemorrhage (PPH): single center experience. Obstet Gynecol 2021;224(2):S501-S501.

(17) Barinov S, Medyannikova I, Borisova A, Tyrskaya Y, Savelieva I, Shamina I, Lazareva O, Kadtsyna T. The Usefulness of Zhukovsky Double Balloon in Obstetric Hemorrhage. Maternal-Fetal Medicine (2019) 1:1.

(18) Kaya B, Tuten A, Guralp O. The Bakri balloon implementation during cesarean section without switching to the lithotomy position. Case Reports in Perinatal Medicine 2016;5(2):81-84.

(19) Escobar MF, Suso JP, Hincapié MA, Echavarría MP, Fernández P, Carvajal J. Experience of combined use of a Bakri uterine balloon and a non-pneumatic anti-shock garment in a university hospital in Colombia. Int J Gynaecol Obstet 2019 Aug;146(2):244-249.

(20) Dalia Y, Agrawal M, Sharma A. Various Modifications of Condom Balloon Tamponade and their Method, Efficacy, Outcomes in Management of Atonic Postpartum Hemorrhage in Tertiary Care Centre- A Observational Study. JMSCR 2018;06(05).

(21) Kong CW, To WWK. Menstrual and reproductive outcomes after use of balloon tamponade for severe postpartum hemorrhage. BMC Pregnancy Childbirth 2018 Nov 21;18(1):451-018-2085-6.

(22) Mattern J, Sibiude J, Picone O, Mandelbrot L. Consequences of Bakri intra-uterine tamponade balloon use in postpartum hemorrhage: Before and after study in a centre without embolisation. Gynecologie, Obstetrique, Fertilite & Senologie 2020.
