# Supplementary material for: Effectiveness of uterine tamponade devices for refractory postpartum haemorrhage after vaginal birth: a systematic review
Source: BJOG. 2021 Jul 19;128(11):1732–43. doi: 10.1111/1471-0528.16819 (PMC9292664; doi:10.1111/1471-0528.16819)
Supplement: Supplementary file 6 — Table S5. Overall methodological quality of included RCTs and non‐RCTs. [file BJO-128-1732-s003.docx]

**Table S5: Overall methodological quality of included RCTs and non-RCTs**

| Randomised studies | | | | | | | |
| --- | --- | --- | --- | --- | --- | --- | --- |
| **Anger 2019** | | | **Bias** | **Authors' judgement** | | **Support for judgement** | |
|  |  |  | Random sequence generation (selection bias) | Unclear | | Method not specified. | |
|  |  |  | Allocation concealment (selection bias) | Low risk | | Not applicable to a cluster stepped wedge trial. | |
|  |  |  | Blinding of participants and personnel (performance bias) | High risk | | Although, outcomes are not likely to be influenced by unblinded participants, unblinded providers might influence them. It is possible that unblinded personnel introduce performance bias by affecting clinical decisions and outcomes. | |
|  |  |  | Blinding of outcome assessment (detection bias) | High risk | | Blinding of outcome assessment is not described. It is possible that unblinded providers have biased the outcomes assessments. Some components of the composite outcomes are likely to be influenced by unblinded personnel, such as compressive sutures and artery ligation, which are more frequently observed than other outcomes such as hysterectomy and maternal death. These last two outcomes are less likely to be influenced by detection bias. | |
|  |  |  | Incomplete outcome data (attrition bias) | Low risk | | No loss of follow-up. | |
|  |  |  | Selective reporting (reporting bias) | Unclear risk | | This trial was registered NCT02910310. The majority of the reported outcomes were described in the study protocol. However, treatment of postpartum infection and level of pain experienced by women were not reposted in the study publication. | |
|  |  |  | Other bias | Low risk | | No | |
| **Dumont 2017** | | **Bias** | | **Authors' judgement** | | **Support for judgement** | |
|  |  | Random sequence generation (selection bias) | | Low risk | | A computer-generated randomization sequence was generated by the principal investigator (AD) and stratified by health centers. Within the strata, women with PPH were individually allocated by clock randomization. | |
|  |  | Allocation concealment (selection bias) | | High risk | | The randomization code was only known by the principal investigator (AD), the project manager (CB) and both local trial supervisors  (not involved in patient care) in Benin and Mali. The trial supervisor was called by phone by the caregiver (a midwife or a doctor) to review inclusion and exclusion criteria for individual possible participants. It is not clear if allocation was concealed by the supervisor; if not, this could have introduced a selection bias. | |
|  |  | Blinding of participants and personnel (performance bias) | | High risk | | Although outcomes are not likely to be influenced by unblinded participants, unblinded personnel might influence them, mainly because blood loss was assessed by visual estimation. Knowing the intervention might affect blood loss and introduce performance bias. | |
|  |  | Blinding of outcome assessment (detection bias) | | High risk | | Outcomes seemed to have been assessed by the same provider that could have been influenced by performance bias. | |
|  |  | Incomplete outcome data (attrition bias) | | Low risk | | No patients were lost from the randomization to the final follow-up by phone, except seven women who died before hospital discharge. | |
|  |  | Selective reporting (reporting bias) | | Low risk | | The reported effect estimate was unlikely to be selected based on the results. The previously published protocol is aligned with the reported outcomes. The study was registered ISRCTN01202389 and outcomes in the protocol and the publication are the same. | |
|  |  | Other bias | | High risk | | Unbalanced in baseline characteristics related to estimated blood loss is a major concern. Estimated blood loss ≥1000 mL was 42% in the intervention group versus 23% in the control group. Reported risk ratios were not adjusted by estimated blood loss at baseline at the analysis. | |
| **Non-randomised studies** | | | | | | | |
| **Laas 2012** | **Bias** | | | | **Authors' judgement** | | **Support for judgement** |
|  | Bias due to confounding | | | | High risk | | Not reported. |
|  | Bias in selection of participants into the study | | | | Low risk | | This is an acute event and the follow-up. |
|  | Bias in classification of interventions | | | | Low risk | | Intervention groups clearly defined: Bakri vs. standard of care. |
|  | Bias due to deviations from intended interventions | | | | Low risk | | In the post-Bakri period, 31/35 vaginal deliveries received Bakri. |
|  | Bias due to missing data | | | | Low risk | | No missing data. |
|  | Bias in measurement of outcomes | | | | Low risk | |  |
|  | Bias in selection of the reported result | | | | Low risk | |  |
|  | Overall bias | | | | High risk | |  |
| Revert  2018 | Bias due to confounding | | | | Low risk | | There is a description of adjustment by confounders; however, there is not a description of stratification nor control of interactions due to the effect modifiers. The authors used a multivariate regression. We assumed that that the medical charts contained valid information There were no post-interventions. There was no time-varying confounding |
|  | Bias in selection of participants into the study | | | | Low risk | | This population-based retrospective cohort study included all women who gave birth in two perinatal networks in France during 2011 and 2012. This is an acute event and the follow-up was retrospective. |
|  | Bias in classification of interventions | | | | Low risk | | Intervention groups clearly defined: Bakri or ebb vs. medical treatment and not affected by the knowledge of the outcome or risk of the outcome. |
|  | Bias in classification of interventions | | | | Low risk | | There were no deviations from the intended intervention beyond what would be expected in usual practice |
|  | Bias due to deviations from intended interventions | | | | Low risk | | Missing was only 0.05% |
|  | Bias due to missing data | | | | Low risk | | The outcome measure was not influenced by knowledge of the intervention received. |
|  | Bias in measurement of outcomes | | | | Low risk | | The methods of outcome assessment were comparable across intervention groups. |
