# Supplementary material for: Effectiveness of uterine tamponade devices for refractory postpartum haemorrhage after vaginal birth: a systematic review
Source: BJOG. 2021 Jul 19;128(11):1732–43. doi: 10.1111/1471-0528.16819 (PMC9292664; doi:10.1111/1471-0528.16819)
Supplement: Supplementary file 7 — Appendix S1. Search strategy. [file BJO-128-1732-s006.docx]

Appendix S1: SEARCH STRATEGY

PUBMED DATABASE

| **Concept** | **Search string** |
| --- | --- |
| #1 - | "Uterine Inertia/therapy"[Mesh] OR "Uterine Balloon Tamponade"[Mesh] |
| #2 - | ("Catheters, Indwelling"[Mesh] OR "Condoms"[Mesh] OR  "Gloves, Surgical"[Mesh] OR "Balloon Occlusion"[Mesh] OR "Pressure"[Mesh] OR UBT [TIAB] OR condom*[TW] OR balloon*[TW] OR glove*[TW] OR “vacuum induced” [TW] OR retraction[TW] OR Bakri [TIAB] OR sengstaken[TIAB] OR Rusch [TIAB] OR  “foley catheter” [TiAB] OR “vacuum force” [TW] OR inpress[TW] OR packing [TW] OR tamponade [TW] OR compression [TW] OR "hemostatic techniques"[TW]) AND (“POSTPARTUM HEMORRHAGE"[Mesh] OR  “UTERINE HEMORRHAGE"[Mesh] OR  “CESAREAN SECTION "[Mesh] OR “PLACENTA PREVIA"[Mesh] OR “UTERINE INVERSION"[Mesh] OR “PLACENTA ACCRETA"[Mesh] OR “POSTPARTUM PERIOD"[Mesh] OR “PREGNANCY OUTCOME"[Mesh] OR “UTERINE INERTIA"[Mesh]  OR “ABRUPTIO PLACENTAE” [Mesh] OR "Pregnancy Complications, Hematologic"[Mesh] OR "Uterine Artery Embolization"[Mesh] OR "Uterine Contraction"[Mesh] OR uterine atony [TW] ) |
| #3 – | (“UTERUS"[Mesh] OR “CERVIX UTERI”[Mesh] OR “PELVIC FLOOR”[Mesh] OR "Delivery, Obstetric"[Mesh] OR   "Labor, Induced"[Mesh] OR "Labor, Obstetric"[Mesh] OR  “pregnancy COMPLICATIONS”[Mesh] OR uterine[TW] OR placenta[TW] OR  “OBSTETRIC LABOR” [TW]  OR postpartum[TW] OR  cesarean[TW] OR caesarean [TW] OR  “pregnancy complication” [TW] OR “pregnancy complications” [TW]) AND ("Hemorrhage"[Mesh] OR “blood loss, surgical” [MH] OR "Hemostatic Techniques"[Mesh] OR "Embolization, Therapeutic"[Mesh] OR "hemostatic techniques"[TW] OR bleeding[TW]  OR Hemorrhag*[TW]  OR haemorrhag*[TW]   OR “blood loss” [TW] OR atonic [TW] OR atony[TW] OR “loss of blood”[TW] ) AND ("Catheters, Indwelling"[Mesh] OR "Condoms"[Mesh] OR  "Gloves, Surgical"[Mesh] OR "Balloon Occlusion"[Mesh] OR "Pressure"[Mesh] OR condom*[TW] OR balloon*[TW] OR glove*[TW] OR “vacuum induced” [TW] OR retraction[TW] OR Bakri [TIAB] OR sengstaken[TIAB] OR Rusch [TIAB] OR  “foley catheter “ [TiAB] OR “vacuum force” [TW] OR inpress[TW] OR packing [TW] OR tamponade [TW] OR Compression[TW] OR UBT [TIAB]) |
| #4- | 1 OR 2 OR 3 |
|  | **NOT ("Animals"[Mesh] NOT ("Animals"[Mesh] AND "Humans"[Mesh]))** |
| #4-Date Filter |  |

EMBASE DATABASE

| **Concept** | **Search string** |
| --- | --- |
| **#1-** | 'intrauterine balloon'/exp |
| **#2**- | ('catheters'/exp OR 'condom'/exp OR 'surgical glove'/exp OR 'balloon occlusion'/exp OR 'occlusion balloon catheter'/exp OR 'pressure'/exp OR UBT:ti,ab OR condom*:ti,ab,de OR balloon*:ti,ab,de OR glove*:ti,ab,de OR "vacuum induced":ti,ab,de OR retraction:ti,ab,de OR Bakri:ti,ab OR sengstaken:ti,ab OR Rusch:ti,ab OR "foley catheter":ti,ab OR "vacuum force":ti,ab,de OR inpress:ti,ab,de OR packing:ti,ab,de OR tamponade:ti,ab,de OR compression:ti,ab,de OR "hemostatic techniques":ti,ab,de) AND ('postpartum hemorrhage'/exp OR 'uterus bleeding'/exp OR 'placenta previa'/exp OR 'uterus inversion'/exp OR 'placenta accreta'/exp OR 'solutio placentae'/exp OR 'uterine artery embolization'/exp OR 'uterus contraction'/exp OR "uterine atony":ti,ab,de OR “atonic uterus”:ti,ab,de OR “postpartum hemorrhage”:ti,ab,de OR “postpartum haemorrhage”:ti,ab,de OR “labor complication”:ti,ab,de OR “delivery complication”:ti,ab,de OR “labour complication”:ti,ab,de) |
| **#3-** | ('uterus'/exp OR 'uterine cervix'/exp OR 'pelvis floor'/exp OR 'obstetric delivery'/exp OR 'labor induction'/exp OR 'labor'/exp OR 'pregnancy complication'/exp OR uterine:ti,ab,de OR placenta:ti,ab,de OR "OBSTETRIC LABOR":ti,ab,de OR postpartum:ti,ab,de OR cesarean:ti,ab,de OR caesarean:ti,ab,de OR "pregnancy complication":ti,ab,de OR "pregnancy complications":ti,ab,de) AND ('bleeding'/exp OR 'operative blood loss'/exp OR 'obstetric hemorrhage'/exp OR 'artificial embolization'/exp OR "hemostatic techniques":ti,ab,de OR bleeding:ti,ab,de OR Hemorrhag*:ti,ab,de OR haemorrhag*:ti,ab,de OR "blood loss":ti,ab,de OR atonic:ti,ab,de OR atony:ti,ab,de OR "loss of blood":ti,ab,de) AND ('catheters'/exp OR 'condom'/exp OR 'surgical glove'/exp OR 'balloon occlusion'/exp OR 'occlusion balloon catheter'/exp OR 'pressure'/exp OR condom*:ti,ab,de OR balloon*:ti,ab,de OR glove*:ti,ab,de OR "vacuum induced":ti,ab,de OR retraction:ti,ab,de OR Bakri:ti,ab OR sengstaken:ti,ab OR Rusch:ti,ab OR "foley catheter":ti,ab OR "vacuum force":ti,ab,de OR inpress:ti,ab,de OR packing:ti,ab,de OR tamponade:ti,ab,de OR Compression:ti,ab,de OR UBT:ti,ab) |
| **#4 –** | 1 OR 2 OR 3 |
| **#5 -** Exclude Publication types | NOT ('letter'/it OR 'editorial'/it OR 'note'/it) |
|  |  |

Cinahl DATABASE

| **Concept** | **Search string** |
| --- | --- |
| **#1 -** | (MH "Uterine Inertia/TH") OR (MH "Uterine Balloon Tamponade+") |
| **#2 -** | ((MH "Catheters+") OR (MH "Condoms+") OR (MH "Gloves+") OR TI UBT OR AB UBT OR condom* OR balloon* OR glove* OR "vacuum induced" OR retraction OR TI Bakri OR AB Bakri OR TI sengstaken OR AB sengstaken OR TI Rusch OR AB Rusch OR TI "foley catheter" OR AB "foley catheter" OR "vacuum force" OR inpress OR packing OR tamponade OR compression OR "hemostatic techniques") AND ((MH "POSTPARTUM HEMORRHAGE+") OR (MH "UTERINE HEMORRHAGE+") OR (MH "CESAREAN SECTION +") OR (MH "PLACENTA PRAEVIA+") OR (MH "UTERINE INVERSION+") OR (MH "PLACENTA ACCRETA+") OR (MH "POSTNATAL PERIOD+") OR (MH "PREGNANCY OUTCOMES+") OR (MH "UTERINE INERTIA+") OR (MH "ABRUPTIO PLACENTAE +") OR (MH "Pregnancy Complications, Hematologic+") OR (MH "Uterine Artery Embolization+") OR (MH "Uterine Contraction+") OR "uterine atony") |
| **#3**- | ((MH "UTERUS+") OR (MH "CERVIX+") OR (MH "Delivery, Obstetric+") OR (MH "Labor, Induced+") OR (MH "pregnancy COMPLICATIONS+") OR uterine OR placenta OR "OBSTETRIC LABOR" OR postpartum OR cesarean OR caesarean OR "pregnancy complication" OR "pregnancy complications") AND ((MH "Hemorrhage+") OR (MH "blood loss, surgical +") OR (MH "Hemostatic Techniques+") OR (MH "Embolization, Therapeutic+") OR "hemostatic techniques" OR bleeding OR Hemorrhag* OR haemorrhag* OR "blood loss" OR atonic OR atony OR "loss of blood") AND ((MH "Catheters+") OR (MH "Condoms+") OR (MH "Gloves+") OR condom* OR balloon* OR glove* OR "vacuum induced" OR retraction OR TI Bakri OR AB Bakri OR TI sengstaken OR AB sengstaken OR TI Rusch OR AB Rusch OR TI "foley catheter" OR AB "foley catheter" OR "vacuum force" OR inpress OR packing OR tamponade OR Compression OR TI UBT OR AB UBT)) |
| **#4-** | #1 OR #2 OR #3 |
| **#5-** |  |

Cochrane DATABASE

| **Concept** | **Search string** |
| --- | --- |
| #1 - | [mh "Uterine Inertia/therapy"] OR [mh "Uterine Balloon Tamponade"] |
| #2 - | ([mh "Catheters, Indwelling"] OR [mh Condoms] OR [mh "Gloves, Surgical"] OR [mh "Balloon Occlusion"] OR [mh Pressure] OR UBT:ti,ab OR condom*:ti,ab,kw OR balloon*:ti,ab,kw OR glove*:ti,ab,kw OR "vacuum induced":ti,ab,kw OR retraction:ti,ab,kw OR Bakri:ti,ab OR sengstaken:ti,ab OR Rusch:ti,ab OR "foley catheter":ti,ab OR "vacuum force":ti,ab,kw OR inpress:ti,ab,kw OR packing:ti,ab,kw OR tamponade:ti,ab,kw OR compression:ti,ab,kw OR "hemostatic techniques":ti,ab,kw) AND ([mh "POSTPARTUM HEMORRHAGE"] OR [mh "UTERINE HEMORRHAGE"] OR [mh "CESAREAN SECTION"] OR [mh "PLACENTA PREVIA"] OR [mh "UTERINE INVERSION"] OR [mh "PLACENTA ACCRETA"] OR [mh "POSTPARTUM PERIOD"] OR [mh "PREGNANCY OUTCOME"] OR [mh "UTERINE INERTIA"] OR [mh "ABRUPTIO PLACENTAE"] OR [mh "Pregnancy Complications, Hematologic"] OR [mh "Uterine Artery Embolization"] OR [mh "Uterine Contraction"] OR "uterine atony":ti,ab,kw) |
| #3 – | ([mh UTERUS] OR [mh "CERVIX UTERI"] OR [mh "PELVIC FLOOR"] OR [mh "Delivery, Obstetric"] OR [mh "Labor, Induced"] OR [mh "Labor, Obstetric"] OR [mh "pregnancy COMPLICATIONS"] OR uterine:ti,ab,kw OR placenta:ti,ab,kw OR "OBSTETRIC LABOR":ti,ab,kw OR postpartum:ti,ab,kw OR cesarean:ti,ab,kw OR caesarean:ti,ab,kw OR "pregnancy complication":ti,ab,kw OR "pregnancy complications":ti,ab,kw) AND ([mh Hemorrhage] OR [mh "blood loss, surgical"] OR [mh "Hemostatic Techniques"] OR [mh "Embolization, Therapeutic"] OR "hemostatic techniques":ti,ab,kw OR bleeding:ti,ab,kw OR Hemorrhag*:ti,ab,kw OR haemorrhag*:ti,ab,kw OR "blood loss":ti,ab,kw OR atonic:ti,ab,kw OR atony:ti,ab,kw OR "loss of blood":ti,ab,kw) AND ([mh "Catheters, Indwelling"] OR [mh Condoms] OR [mh "Gloves, Surgical"] OR [mh "Balloon Occlusion"] OR [mh Pressure] OR condom*:ti,ab,kw OR balloon*:ti,ab,kw OR glove*:ti,ab,kw OR "vacuum induced":ti,ab,kw OR retraction:ti,ab,kw OR Bakri:ti,ab OR sengstaken:ti,ab OR Rusch:ti,ab OR "foley catheter":ti,ab OR "vacuum force":ti,ab,kw OR inpress:ti,ab,kw OR packing:ti,ab,kw OR tamponade:ti,ab,kw OR Compression:ti,ab,kw OR UBT:ti,ab) |
| #4- | 1 OR 2 OR 3 |
|  | **Central Trials Registry only** |
| #4-Date Filter |  |

LILACS DATABASE

| **Concept** | **Search string** |
| --- | --- |
| #1 - | (MH: (Uterine Inertia/TH)) OR (MH: (Uterine Balloon Tamponade)) |
| #2 - | ((MH: (Catheters, Indwelling)) OR (MH: (Condoms)) OR (MH: (Gloves, Surgical)) OR (MH: (Balloon Occlusion)) OR (MH: (Pressure)) OR (TI: (UBT)) OR (AB: (UBT)) OR condom* OR balloon* OR glove* OR (vacuum induced) OR retraction OR (TI: (Bakri)) OR (AB: (Bakri)) OR (TI: (sengstaken)) OR (AB: (sengstaken)) OR (TI: (Rusch)) OR (AB: (Rusch)) OR (TI: (foley catheter)) OR (AB: (foley catheter)) OR (vacuum force) OR inpress OR packing OR tamponade OR compression OR (hemostatic techniques)) AND ((MH: (POSTPARTUM HEMORRHAGE)) OR (MH: (UTERINE HEMORRHAGE)) OR (MH: (CESAREAN SECTION )) OR (MH: (PLACENTA PREVIA)) OR (MH: (UTERINE INVERSION)) OR (MH: (PLACENTA ACCRETA)) OR (MH: (POSTPARTUM PERIOD)) OR (MH: (PREGNANCY OUTCOME)) OR (MH: (UTERINE INERTIA)) OR (MH: (ABRUPTIO PLACENTAE )) OR (MH: (Pregnancy Complications, Hematologic)) OR (MH: (Uterine Artery Embolization)) OR (MH: (Uterine Contraction)) OR (uterine atony)) |
| #3 – | ((MH: (UTERUS)) OR (MH: (CERVIX UTERI)) OR (MH: (PELVIC FLOOR)) OR (MH: (Delivery, Obstetric)) OR (MH: (Labor, Induced)) OR (MH: (Labor, Obstetric)) OR (MH: (pregnancy COMPLICATIONS)) OR uterine OR placenta OR (OBSTETRIC LABOR) OR postpartum OR cesarean OR caesarean OR (pregnancy complication) OR (pregnancy complications)) AND ((MH: (Hemorrhage)) OR (MH: (blood loss, surgical)) OR (MH: (Hemostatic Techniques)) OR (MH: (Embolization, Therapeutic)) OR (hemostatic techniques) OR bleeding OR Hemorrhag* OR haemorrhag* OR (blood loss) OR atonic OR atony OR (loss of blood)) AND ((MH: (Catheters, Indwelling)) OR (MH: (Condoms)) OR (MH: (Gloves, Surgical)) OR (MH: (Balloon Occlusion)) OR (MH: (Pressure)) OR condom* OR balloon* OR glove* OR (vacuum induced) OR retraction OR (TI: (Bakri)) OR (AB: (Bakri)) OR (TI: (sengstaken)) OR (AB: (sengstaken)) OR (TI: (Rusch)) OR (AB: (Rusch)) OR (TI: (foley catheter)) OR (AB: (foley catheter)) OR (vacuum force) OR inpress OR packing OR tamponade OR compression OR (TI: (UBT)) OR (AB: (UBT))) |
| #4 –  #1 OR #2 OR #3 | ((MH: (Uterine Inertia/TH)) OR (MH: (Uterine Balloon Tamponade))) OR  (((MH: (Catheters, Indwelling)) OR (MH: (Condoms)) OR (MH: (Gloves, Surgical)) OR (MH: (Balloon Occlusion)) OR (MH: (Pressure)) OR (TI: (UBT)) OR (AB: (UBT)) OR condom* OR balloon* OR glove* OR (vacuum induced) OR retraction OR (TI: (Bakri)) OR (AB: (Bakri)) OR (TI: (sengstaken)) OR (AB: (sengstaken)) OR (TI: (Rusch)) OR (AB: (Rusch)) OR (TI: (foley catheter)) OR (AB: (foley catheter)) OR (vacuum force) OR inpress OR packing OR tamponade OR compression OR (hemostatic techniques)) AND ((MH: (POSTPARTUM HEMORRHAGE)) OR (MH: (UTERINE HEMORRHAGE)) OR (MH: (CESAREAN SECTION )) OR (MH: (PLACENTA PREVIA)) OR (MH: (UTERINE INVERSION)) OR (MH: (PLACENTA ACCRETA)) OR (MH: (POSTPARTUM PERIOD)) OR (MH: (PREGNANCY OUTCOME)) OR (MH: (UTERINE INERTIA)) OR (MH: (ABRUPTIO PLACENTAE )) OR (MH: (Pregnancy Complications, Hematologic)) OR (MH: (Uterine Artery Embolization)) OR (MH: (Uterine Contraction)) OR (uterine atony))) OR  (((MH: (UTERUS)) OR (MH: (CERVIX UTERI)) OR (MH: (PELVIC FLOOR)) OR (MH: (Delivery, Obstetric)) OR (MH: (Labor, Induced)) OR (MH: (Labor, Obstetric)) OR (MH: (pregnancy COMPLICATIONS)) OR uterine OR placenta OR (OBSTETRIC LABOR) OR postpartum OR cesarean OR caesarean OR (pregnancy complication) OR (pregnancy complications)) AND ((MH: (Hemorrhage)) OR (MH: (blood loss, surgical)) OR (MH: (Hemostatic Techniques)) OR (MH: (Embolization, Therapeutic)) OR (hemostatic techniques) OR bleeding OR Hemorrhag* OR haemorrhag* OR (blood loss) OR atonic OR atony OR (loss of blood)) AND ((MH: (Catheters, Indwelling)) OR (MH: (Condoms)) OR (MH: (Gloves, Surgical)) OR (MH: (Balloon Occlusion)) OR (MH: (Pressure)) OR condom* OR balloon* OR glove* OR (vacuum induced) OR retraction OR (TI: (Bakri)) OR (AB: (Bakri)) OR (TI: (sengstaken)) OR (AB: (sengstaken)) OR (TI: (Rusch)) OR (AB: (Rusch)) OR (TI: (foley catheter)) OR (AB: (foley catheter)) OR (vacuum force) OR inpress OR packing OR tamponade OR compression OR (TI: (UBT)) OR (AB: (UBT)))) |
